# Effect of three common IL-17 single nucleotide polymorphisms on the risk of developing gastric cancer

**DOI:** 10.3892/ol.2014.2827

**Published:** 2014-12-23

**Authors:** YA-WEN GAO, MEILI XU, YAN XU, DAN LI, SHENGHUA ZHOU

**Affiliations:** 1 Department of Gerontology, The Second Xiangya Hospital, Central South University, Changsha, Hunan 410011, P.R. China; 2 Department of Vasculocardiology, The Second Xiangya Hospital, Central South University, Changsha, Hunan 410011, P.R. China

**Keywords:** IL-17, gastric cancer, polymorphism

## Abstract

A 1:1 matched case-control study was conducted to analyze the association between three common interleukin (IL)-17A and IL-17F single nucleotide polymorphisms (SNPs) and the risk of developing gastric cancer. Genotyping of SNPs rs2275913, rs763780 and rs3748067 within the IL-17 gene were detected by performing polymerase chain reaction-restriction fragment length polymorphism analysis. Gastric cancer patients were more likely to be cigarette smokers, alcohol drinkers and have a family history of cancer in their first-degree relatives. Patients carrying the rs763780 polymorphism were correlated with a significant increased risk of gastric cancer in codominant, dominant and recessive models. Additionally, individuals with the rs763780 polymorphism were correlated with a markedly increased risk of gastric cancer among alcohol drinkers in codominant, dominant and recessive models. Furthermore, a significant correlation was identified between the rs763780 polymorphism and the consumption of alcohol. However, no association was identified between rs2275913 and rs3748067 polymorphisms and the risk of developing gastric cancer. Thus, the present study reported that the rs763780 polymorphism may be associated with risk of developing gastric cancer in the population studied, particularly in alcohol drinkers.

## Introduction

Gastric cancer is one of the most common types of cancer and is associated with a high incidence of mortality worldwide (1,2). Although genetic and environmental factors in addition to *Helicobacter pylori* infections have been identified to be important in the development of gastric cancer (3–5), the precise etiology of the disease remains unclear. Numerous studies have reported that inflammation-associated gene polymorphisms may be involved in the development of gastric cancer, including tumor necrosis factor α and interleukin (IL) genes (6–8).

IL-17 is a cytokine that is secreted exclusively by activated T cells, which bridge the adaptive and innate immune systems (9,10). IL-17A and IL-17F are important members of the IL-17 cytokine family; they are preferentially produced by T helper 17 (Th17) cells, which are responsible for the pathogenic activity of the lineage of cluster of differentiation (CD)4^+^ effector cells and multiple proinflammatory mediators (11,12). A previous study reported that IL-17A and IL-17F single nucleotide polymorphisms (SNPs) are associated with the risk of developing gastric cancer (13). However, subsequent replication studies investigating the association between IL-17A and IL-17F variants with the risk of developing gastric cancer were controversial (10,14–16). This discrepancy may be attributed to the relatively small sample size of previous studies and the genetic heterogeneity of polymorphisms in gastric cancer among different populations.

Therefore, to clarify the conflicting findings of previous reports, the present study used multiple genetic statistical models to conduct a 1:1 matched case-control study to analyze the association between three common IL-17A and IL-17F SNPs and the risk of developing gastric cancer in the study population.

## Materials and methods

### Study population

Between May 2010 and May 2012, 572 gastric cancer patients were recruited from the Second Xiangya Hospital of Central South University (Changsha, China); the gastric cancer patients were recently and histopathologically diagnosed with primary gastric cancer. The exclusion criteria were as follows: Patients who exhibited secondary or recurrent tumors, a history of other malignant neoplasm, or inadequate organ function. Gender and age-matched (±5 years) individuals were selected from those who visited the Second Xiangya Hospital for a routine health check-up. Additionally, 572 controls were selected from inpatients in the Departments of Orthopedics and Dermatology, and were matched to the selected gastric cancer patients by age (±5 years) and gender. None of the control patients had a history of cancer. Written informed consent was obtained from all of the patients prior to participation in the present study and the protocol of the present study was approved by the ethics committee of the Second Xiangya Hospital of Central South University.

A self-designed questionnaire was developed to investigate the demographic characteristics of all case and control patients. All of the patients completed the questionnaire, which was conducted by trained interviewers who were not aware of the study hypothesis. *H. pylori* infection status was evaluated using histological examination or a rapid urea breath test; if one of the tests indicated a positive result, the patients was diagnosed as positive for *H. pylori* infection.

### Genotype analysis

All of the study participants provided a 5-ml venous blood sample, which was stored at −20°C until required. According to the manufacturer’s instructions, genomic DNA was extracted from the peripheral venous blood samples using the TIANamp blood DNA kit (Tiangen Biotech Co., Ltd., Beijing, China). Genotyping of SNPs rs2275913, rs3748067 and rs763780 within the IL-17 gene were detected by performing polymerase chain reaction-restriction fragment length of polymorphism (PCR-RFLP) analysis. The primers used for SNPs rs2275913, rs3748067 and rs763780 were designed using MassARRAY^®^ Assay Design software version 3.1 (Sequenom, San Diego, CA, USA) according to the manufacturer’s instructions (Table I). The cycling program involved preliminary denaturation at 95°C for 5 min, followed by 35 cycles of denaturation at 95°C for 30 sec, annealing at 62°C for 30 sec, 72°C for 30 sec and a final extension at 72°C for 10 min. The digested PCR products were run on a 2% agarose gel stained with ethidium bromide and ultraviolet light, followed by sequencing of the PCR products using an automated sequencing system (PTC-200 DNA Engine PThermal Cycler; MJ Research, Inc., Waltham, MA, USA).

### Statistical analysis

Continuous variables are presented as the mean ± standard deviation and were analyzed using the independent sample Student’s t-test. Categorical variables are presented as frequencies (percentages) and were analyzed using the χ^2^ test. The Hardy-Weinberg equilibriums between groups were compared using the χ^2^ test. A conditional multiple logistical regression model was used to assess the effects of SNPs rs2275913, rs3748067 and rs763780 on gastric cancer risk; the results were adjusted for potential confounding variables prior to their expression as odds ratios (ORs) and 95% confidence intervals (CIs). The homozygous genotypes of the three SNPs were used as the reference group. All P-values were two sided, and P<0.05 was considered to indicate a statistically significant difference. All statistical analyses were performed using SPSS software, version 11.0 (SPSS, Inc., Chicago, IL, USA) for Windows.

## Results

### Characteristics of patients and controls

The characteristics of the case and control patients are demonstrated in Table II. As expected, no significant difference was identified between the case and control patients in terms of gender or age (P>0.05). The gastric cancer cases were more likely to be cigarette smokers, alcohol drinkers and have a history of cancer in their first-degree relatives. However, no significant difference was identified in the gender, age, alcohol consumption or annual income between the case and control patients.

### Multivariate analysis

The genotype distributions of IL-17 rs2275913 and rs763780 among the controls were within the parameters of the Hardy-Weinberg equilibrium; however, the distributions of IL-17 rs3748067 were not. Using conditional regression analysis, it was identified that individuals carrying the IL-17 rs763780 CC genotype demonstrated a marginally increased risk of developing gastric cancer when compared with the TT genotype in the codominant model, with an adjusted OR (95% CI) of 2.27 (1.51–3.45) (Table III). Furthermore, the present study identified that individuals carrying the rs763780 polymorphism were correlated with a significantly increased risk of developing gastric cancer in dominant and recessive models, demonstrating an OR (95% CI) of 1.71 (1.27–2.30) and 2.20 (1.47–3.34), respectively. However, no significant association was observed between rs2275913 and rs3748067 polymorphisms, and the risk of developing gastric cancer.

### Association between IL-17 genotypes and environmental factors

Additionally, the present study investigated the interaction between the rs763780 polymorphism with the risk of developing gastric cancer via cigarette smoking, alcohol consumption and *H. pylori* infection (Table IV). Individuals with the rs763780 TT genotype who were alcohol drinkers were significantly associated with an increased risk of developing gastric cancer (OR,4.27; 95% CI, 2.24–8.63). Furthermore, the rs763780 polymorphism was correlated with an increased risk of developing gastric cancer among alcohol drinkers in dominant and recessive models [OR (95%CI), 2.34 (1.47–3.78); OR (95%CI), 4.19 (2.20–8.41), respectively]. Thus, a significant interaction was identified between the rs763780 polymorphism and alcohol consumption in gastric cancer risk (P=0.02). However, no significant interaction was identified between the rs763780 polymorphism and cigarette smoking or *H. pylori* infection in the risk of developing gastric cancer.

## Discussion

Identification of genes involved in the genetic predisposition to or progression of cancer is important in clinical practice and in basic medical research. IL-17A and IL-17F are expressed by Th17 cells and are involved in coordinating local tissue inflammation (17,18). Various studies have demonstrated that IL-17A and IL-17F may be involved in the development of gastric cancer (15,16,19), however, the results were unclear. Therefore, the present study conducted a case-control investigation to provide a more reliable conclusion of the association between the IL-17A and IL-17F SNPs, and gastric cancer.

The rs2275913 polymorphism is located at the 5′ region of the IL-17A gene and, therefore, may regulate gene transcription (20). By contrast, the rs763780 polymorphism is located in the coding region and is a missense mutation, which may influence the protein structure and function (19,21). Hence, the functions of these two SNPs requires further investigation.

IL-17 family members are a cytokines and are involved in coordinating local tissue inflammation by the release of proinflammatory and neutrophil-mobilizing cytokines (21). Polymorphisms in IL-17 cytokines alter the activity of interleukins and may alter cytokine function, thus, dysregulating IL-17 expression (12). Previous studies reported that IL-17 polymorphisms are associated with the risk of developing gastric cancer in Japanese, Chinese and Iranian populations, however, these genetic polymorphisms appear to exhibit different effects in each populations (10,13–16,19). Shibata *et al* (10) reported that IL-17 rs2275913 polymorphism was significantly associated with the development of gastric cancer; however, Wu *et al* (13) reported that while the rs763780 polymorphism was involved in the development of gastric cancer, the rs2275913 polymorphism was not. In the present study, it was identified that the rs763780 polymorphism appears to be associated with the risk of developing gastric cancer, as well as interacting with the variable of alcohol consumption. The discrepancy of the results of the present study and previous studies may be explained by differences in the ethnicities of the participants, the source of the control subjects, the sample size, or by chance. Additional studies are required to clarify the association between IL-17 polymorphisms and the risk of developing gastric cancer.

The present study was limited by a number of factors. First, the study was conducted in a single hospital in Changsha, China; therefore, the participants may not have been representative of the Chinese population as a whole. Second, the etiology of gastric cancer involves multiple genes and environmental factors, which were not considered in the present study. Third, the genotypic distributions of rs3748067 in the case and control patients were not consistent with Hardy-Weinberg equilibriums, which indicates that the cohort of the present study may be a poor representation of the Chinese population. Finally, the predominant limitation of the present study was the relatively small sample size, therefore, additional large sample size studies are required to confirm our results.

In conclusion, the present study reported that the rs763780 polymorphisms may be associated with risk of developing gastric cancer in a Chinese population, particularly in alcohol drinkers. However, no association was identified between the rs2275913 and rs3748067 polymorphisms and the risk of gastric cancer. Therefore, the rs763780 polymorphism may be used as a diagnostic biomarker for gastric cancer. Additional large sample size studies are required to confirm the role of IL-17 polymorphisms in the development of gastric cancer.

## Figures and Tables

**Table I:** Primers and PCR-RFLP analysis for VEGF genetic polymorphisms.

SNP	Primer sequence	Amplification fragment, bp
rs2275913	Forward, 5′-GCCCTTCCCATTTTCCTTCAGA-3′ Reverse, 5′-CCAATCAACTGGGGATGGATGA-3′	210
rs763780	Forward, 5′-CTGTTTCCATCCGTGCAGGTC-3′ Reverse, 5′-TGGTGACTGTTGGCTGCACCT-3′	188
rs3748067	Forward, 5′-AAGCAGGGAGCCTGCAGAGTG-3′ Reverse, 5′-GGCACCACACAACCCAGAAAG-3′	217

PCR-RFLP, polymerase chain reaction-restriction fragment length polymorphism; VEGF, vascular endothelial growth factor; SNP, single nucleotide polymorphism.

**Table II:** Demographic characteristics of included cases and controls.

	Cases (n=572)		Controls (n=572)			
				
Variable	n	%	n	%	*t* or χ^2^	P-value
Age, years						
<55	249	43.53	253	44.23		
≥55	323	56.47	319	55.77	0.06	0.810
Gender						
Female	220	38.46	220	38.46		
Male	352	61.54	352	61.54	0.00	1.000
Cancer history in first-degree relatives						
No	522	91.26	569	99.48		
Yes	50	8.74	3	0.52	43.70	<0.001
Alcohol drinker						
Never	333	58.22	375	65.56		
Ever	239	41.78	197	34.44	6.54	0.010
Cigarette smoker						
Never	386	67.48	424	74.13		
Ever	186	32.52	148	25.87	6.11	0.010
*Helicobacter pylori*						
Negative	201	35.14	308	53.85		
Positive	371	64.86	266	46.50	39.80	<0.001

**Table III:** Genotype frequencies of three SNPs of IL-17A and IL-17F in gastric cancer patients and controls.

	Cases (n=572)		Controls (n=572)			OR (95% CI)		
				
Genotype	n	%	n	%	Hardy-Weinberg equilibrium	Codominant model	Dominant model	Recessive model
rs2275913								
GG	239	41.8	260	45.4		Ref. (1.0)		
GA	250	43.7	241	42.1		1.13 (0.87–1.46)		
AA	83	14.5	72	12.5	0.17	1.25 (0.86–1.83)	1.16 (0.91–1.47)	1.18 (0.83–1.68)
rs763780								
CC	420	73.5	472	82.5		Ref. (1.0)		
CT	67	11.7	58	10.2		1.30 (0.88–1.93)		
TT	85	14.8	42	7.3	0.09	2.27 (1.51–3.45)	1.71 (1.27–2.30)	2.20 (1.47–3.34)
rs3748067								
TT	460	80.4	458	80.4		Ref. (1.0)		
TC	70	12.2	66	11.5		1.06 (0.72–1.54)		
CC	42	7.4	47	8.1	<0.001	0.89 (0.56–1.41)	0.99 (0.90–1.69)	0.90 (0.57–1.42)

SNP, single nucleotide polymorphism; IL, interleukin; OR, odds ratio; CI, confidence interval; Ref. the GG genotype is the reference for the GA genotype.

**Table IV:** Association between rs763780 polymorphism and gastric cancer risk stratified by demographic characteristics.

	Codominant model					Dominant model			Recessive model		
			
	CC		TT			CT + TT			CC + CT		
							
Rs763780 polymorphism	Case	Control	Case	Control	OR (95% CI)	Case	Control	OR (95% CI)	Case	Control	OR (95%CI)
Cigarette smoker											
Never	303	363	38	23	1.98 (1.12–3.56)	83	61	1.63 (1.12–2.39)	348	401	1.90 (1.08–3.41)
Ever	117	109	47	19	2.30 (1.23–4.42)	69	39	1.65 (1.00–2.73)	139	129	2.30 (1.24–4.36)
Alcohol drinker											
Never	265	312	27	28	1.18 (0.64–2.13)	68	63	1.27 (0.85–1.89)	306	347	1.09 (0.61–1.97)
Ever	155	160	58	14	4.27 (2.24–8.63)	84	37	2.34 (1.47–3.78)	181	183	4.19 (2.20–8.41)
*Helicobacter pylori*											
Negative	142	256	34	22	2.79 (1.51–5.20)	59	52	2.05 (1.31–3.20)	167	286	2.65 (1.45–4.91)
Positive	278	216	51	20	1.98 (1.12–3.61)	93	48	1.51 (1.00–2.28)	320	244	1.94 (1.10–3.53)

OR, odds ratio; CI, confidence interval.
